# Maternally Derived Antibodies to Foot-and-Mouth Disease Virus Modulate the Antigenic Specificity of Humoral Responses in Vaccinated Cattle

**DOI:** 10.3390/vaccines11121844

**Published:** 2023-12-13

**Authors:** Jamaliah Senawi, Ginette Wilsden, Clare F. J. Browning, Anna B. Ludi, Mazatonazuar Meor Ismail, Halimah Senin, Simon Gubbins, Donald P. King, David J. Paton

**Affiliations:** 1 Department of Veterinary Services, Putrajaya 62630, Malaysia; 2 The Pirbright Institute, Ash Road, Pirbright GU24 0NF, UKsimon.gubbins@pirbright.ac.uk (S.G.); donald.king@pirbright.ac.uk (D.P.K.)

**Keywords:** FMD vaccination, maternal antibody, antigen specificity, epitope masking

## Abstract

Vaccination is widely used to control foot-and-mouth disease (FMD), but maternal antibodies may interfere with the response to vaccination in calves. This study, conducted on a regularly vaccinated Malaysian dairy farm, aimed to optimise the vaccination regime by measuring the in vitro neutralising virus antibody responses of 51 calves before and after vaccination with a one or two dose vaccination regime starting at 2–7 months old. The presence of maternal antibodies was associated with poor post-vaccination antibody responses after a single dose of vaccine in calves less than 6 months old. However, a second dose of vaccine given three weeks later, improved the antibody responses in all ages of calves. This confirms the view that in regularly vaccinated farms, some combination of delay and revaccination is needed to achieve effective immunization of calves. Sera from cows and pre-vaccinated calves neutralised homologous serotype A vaccine virus more strongly than a heterologous serotype A field virus, but this pattern was reversed in some calves after vaccination. The strength of heterologous responses in calves 49 days after first vaccination correlated to the amount of transferred maternal antibody, suggesting that pre-existing antibodies could have modulated the specificity of these active antibody responses. If confirmed, such an effect by pre-existing antibodies could have wider implications for broadening the coverage of FMD vaccine responses.

## 1. Introduction

Foot-and-mouth disease (FMD) affects cloven-hoofed animals and causes a vesicular disease associated with serious production losses in domestic livestock, especially cattle and pigs [1]. Infection with FMD virus (FMDV) can also cause fatal myocarditis in young stock. The disease is difficult and costly to control and restricts trade of livestock and their products [2]. The causative agent is a Picornavirus that exists as multiple serotypes and strains requiring careful selection of vaccines for antigenic relevance. Vaccination with killed vaccines has contributed to the successful control and eradication of FMD in western Europe and parts of South America [3,4]. However, FMD remains endemic in many African and Asian countries (https://www.woah.org/en/disease/foot-and-mouth-disease/#ui-id-2; accessed on 6 December 2023), where both routine and emergency vaccination are often a cornerstone of control policies [5]. For routine vaccination, target animals should be vaccinated at an early age and regularly boosted to maintain immunity [6]. Two doses of vaccine given about a month apart are often recommended for primary vaccination in endemic settings as the booster dose results in a stronger antibody response and requires a less potent vaccine to provide protection until revaccination, commonly around six months later [7,8,9]. However, this recommendation may be ignored, so that animals receive only a single dose of vaccine when first vaccinated. It is well established that maternally derived antibodies (MDA) to FMDV that are transferred in colostrum from immune dams to their offspring provide protection but interfere with the development of acquired immunity, although this interference varies with differences in the timing of vaccination of dams and especially calves, the potency of vaccines, the nature of the adjuvants within them, the amount and timing of colostral consumption and the different serological tests and test cut-off thresholds used to predict protection [10,11,12,13,14,15,16,17,18]. This creates uncertainty about the effectiveness of FMD vaccination and what will be the most appropriate vaccination regime.

FMD has never been reported in Malaysian Borneo (Sabah and Sarawak) but there has been a long history of FMD in Peninsular Malaysia mainly involving serotypes O and A [19] (https://www.wrlfmd.org/east-and-southeast-asia/malaysia; accessed on 6 December 2023). Consequently, vaccination is widely used to protect animals from disease and to limit virus transmission. Since FMD vaccines differ in potency and in their antigenic match to field viruses, it is recommended to monitor their performance in the field and adjust the vaccination regime according to situation-specific findings [6]. This study was designed to evaluate the immune responses of cattle vaccinated in the field to estimate the protection afforded against a Malaysian field strain of FMDV and to optimise the vaccination regime for calves to minimise interference from MDA in herds where routine prophylactic vaccination is carried out.

## 2. Materials and Methods

### 2.1. Study Location and Sampling

The farm was selected for its favourable location, husbandry practices, and zoo-sanitary control measures. The study was authorised, from an ethical point of view, by the farm manager and the Malaysian Veterinary Authority. The farm was a government-operated cow and calf unit with approximately 500 dairy cattle of Mafriwal and Friesian Shahiwal breed, located in southern Peninsular Malaysia where FMD occurrence is uncommon. All cattle on this farm had been vaccinated for FMD twice a year (January and June), starting at six months of age and clinical cases of FMD had never been reported. However, no regular post-vaccination monitoring program had been carried out on the farm to assess the performance of the FMD vaccination regime.

For this study, a group of 51 calves were selected that had not been previously vaccinated for FMD. Forty-eight out of fifty-one dams (cows) of the selected calves were also included in the study. Blood samples were collected from the cows 56 days after their summer vaccination. The 51 calves, aged between two and seven months, were blood sampled and vaccinated for the first time 56 days after the cows were sampled. Further blood samples were collected from calves 21 and 49 days later. Approximately half of the calves (n = 26) were given a booster vaccination 21 days after their first vaccination. Blood was collected from each cow and calf and separated sera were heated at 56 °C for 30 min and then stored at −20 °C until tested.

### 2.2. Vaccine and Vaccination

The FMD vaccine used was an aqueous polyvalent formulation with a specified potency of at least 6PD_50_ and purchased by the Government of Malaysia for vaccination of ruminants. Cows and calves received a 2 mL dose of vaccine containing the vaccine strains A/May-97, O1 Manisa, O-3039, and Asia 1 Shamir (Aftovaxpur^®^ Merial Animal Health Ltd., Pirbright, UK) administered subcutaneously in the front of the shoulder. The adult cows had been vaccinated between seven times over three years and twenty-two times over ten years. On the days of vaccination and sampling for this study, every cow and calf selected were physically examined to ensure no clinical signs of FMD were present.

### 2.3. Serology

The Merial vaccine had been purified to remove non-structural proteins (NSP) of FMDV and so all the collected sera were tested by ELISA for antibodies to NSP, as an indicator of undisclosed FMDV infection, using the PrioCHECK^®^ FMDV-NS test kit produced by Prionics Lelystad B.V. [20]. Testing and interpretation followed the manufacturer’s instructions, with samples scoring ≥ 50 percent inhibition considered as positive.

The sera were also tested for neutralising antibodies to two strains of serotype A FMDV, including the vaccine strain, A/May-97, and a field isolate, A/MAY/2/2011, chosen as a representative of the A/ASIA/Sea-97 lineage that has circulated in the region. Serological testing was carried out at the FAO World Reference Laboratory for FMD (WRLFMD) at Pirbright using their standard method for the virus neutralisation test (VNT) in which sera are diluted in a twofold series starting at one in four [21]. All sera were tested in batches so that the same serum was tested against the homologous/vaccine virus and heterologous/field isolate simultaneously with the same IB-RS-2 cell suspension. Titres were expressed as log_10_ serum dilutions neutralising 50% of FMDV. The test specificity approaches 100% at a 1.2 log_10_ cut-off and for calves with low VNT titres, r_1_ values were calculated (arithmetic heterologous titre divided by arithmetic homologous titre) with or without exclusion of sera failing to score positive at this threshold to at least one of the viruses under test.

An FMD vaccine with a vaccine potency of ≥6 PD_50_ should protect with a probability of >90% [22]. The relationship between post-vaccination VNT titre and protection, using the WRLFMD method has been analysed for homologous and heterologous protection (i.e., against a strain that is the same or different to that in the vaccine), respectively [23,24]. Accordingly, for serotype A, using the homologous virus in the test, log_10_ titres of 1.4 and 2.1 equate to 50% and 95% probability of homologous protection. Using the heterologous virus in the test, log_10_ titres of between 1.17 and 1.67 were associated with a 75% probability of heterologous protection with different strains of serotype A.

### 2.4. Antigenic Matching

One-way antigenic relationship (r_1_) values between the vaccine virus and the field virus were calculated with each of the sera obtained from the 48 cows and from the 51 calves before and after vaccination. A value of ≥0.3 is considered as indicating an acceptable match [21,25].

### 2.5. Statistical Analysis

The duration of maternally derived antibody was analysed using linear regression implemented in Matlab (version R2020b; The Mathworks Inc., Natick, MA, USA). Differences in the antibody responses of calves in different age groups were assessed using linear mixed models. The response variable was log_10_ titre (either homologous or heterologous) and explanatory variables were age (2, 3, 4, 5, 6, or 7 months old, as a categorical variable), days post vaccination (0, 21, or 49 days, as a categorical variable) and whether or not the calf received a booster vaccination (boosted or not boosted, as a categorical variable) as fixed effects and calf as a random effect. Model selection proceeded by stepwise deletion of non-significant (*p* > 0.05) terms as judged by likelihood ratio tests, starting from a model including all explanatory variables and pairwise interactions between them. The final models are presented in Supplementary Table S1. The analyses were implemented using the nlme package [26] in R (version 4.2.3) [27]. The relationship between the change in homologous titre or the difference in homologous and heterologous titres at 21 and 49 days post-vaccination and the pre-vaccination titre was assessed using Spearman’s rank correlation coefficient. This was chosen to allow for potential non-normality and non-linearity in the data.

## 3. Results

### 3.1. Disease and Infection Status

No signs of FMD were detected and all the sera collected from cows and their calves scored negative for antibodies to FMDV NSP, indicating absence of undisclosed infection.

### 3.2. Virus Neutralisation Tests

All results are provided in Supplementary Table S2.

#### 3.2.1. Homologous Neutralising Antibodies

**Figure 1:**
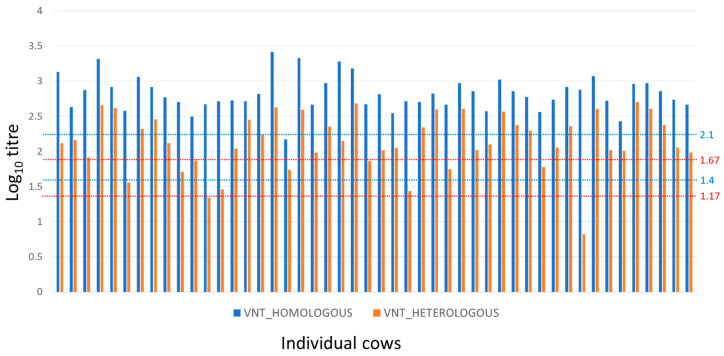
Homologous and heterologous FMDV-serotype A-specific neutralisation antibody titres for cows (dams). Reciprocal neutralisation antibody titres (log_10_) of individual adult cows against homologous (A/May-97, blue) and heterologous (A/MAY/2/2011, red) viruses. Dotted lines provide thresholds predicted for 95% and 50% homologous protection (blue, ref. [23]) and for upper and lower predicted thresholds of 75% heterologous protection (red, ref. [24]).

The results for the adult cow sera are summarised in Figure 1. The titres against the A/May-97 vaccine virus ranged from 2.17 to 3.53 (mean and standard deviation of 2.84 and 0.27) indicating a high probability of homologous protection [23].

**Figure 2:**
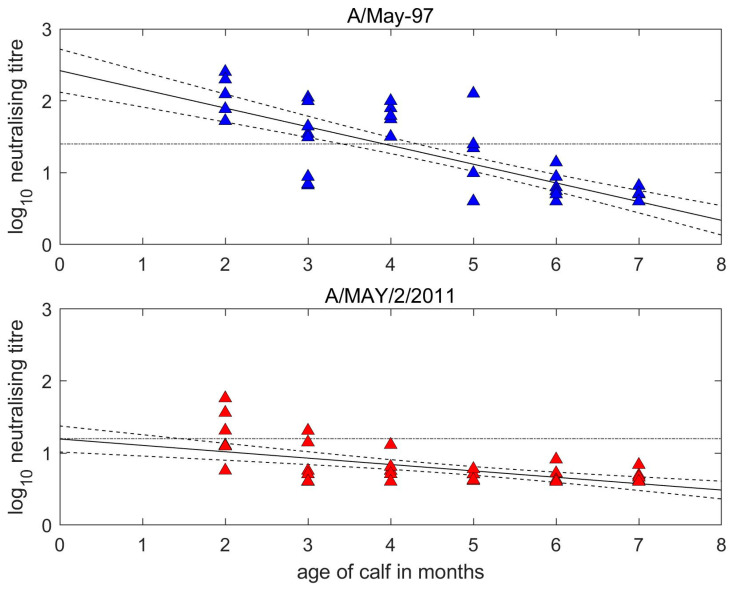
Decay of maternally derived neutralisation titres in unvaccinated calves. Reciprocal log_10_ titres measured against the homologous (A/May-97, blue) vaccine virus and the heterologous (A/MAY/2/2011, red) field virus. Dots indicate actual titres at given calf ages, with predicted decay line and associated uncertainty determined by linear regression. The horizontal dotted black lines represent thresholds for expectancy of protection for serotype A. This is set at 1.4 log_10_ for homologous titres equating to 50% protection [23] and at 1.2 log_10_ for heterologous titres, which is the lower threshold for 75% cross-protection [24].

At the time of first vaccination, the A/May-97 VNT titres of the calves were inversely related to their age (Figure 2). The two-months-old and seven-months-old calves had titres against the vaccine strains of 1.8–2.3 log_10_ and <0.6 log_10_, respectively. The estimated half-life for MDA was 1.16 (95% confidence interval (CI): 0.95–1.47) months and predicted 50% protection against homologous FMDV challenge was lost at 3.92 (95% CI: 3.41–4.32) months of age (based on antibody titres of 1.4 log_10_ being protective) [23]. Homologous antibodies became undetectable (<0.6) at 6.99 (95% CI: 6.47–7.72) months of age.

**Figure 3:**
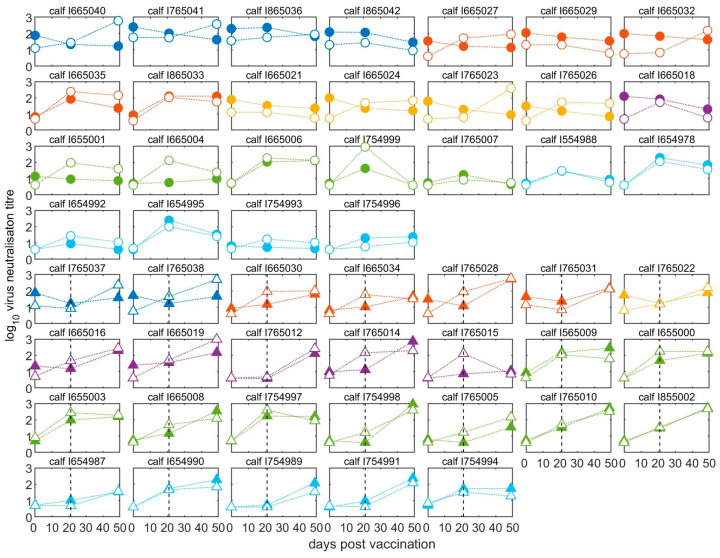
Neutralising antibodies in calves before and after vaccination. All calves were vaccinated for the first time at day 0 and half were revaccinated (boosted) at day 21. Solid points represent homologous (A/May/97) titres, whilst open points represent heterologous (A/MAY/2/2011) titres. Circles represent unboosted calves (top four rows) and triangles represent calves given a booster vaccination at 21 dpv (bottom four rows). The black dashed line indicates the time of the booster vaccination. Samples were taken on the day of first vaccination and then at 21 and 49 days afterwards. Age of calves: 2 months, dark blue; 3 months, red; 4 months, yellow; 5 months, purple; 6 months green; 7 months, light blue.

**Figure 4:**
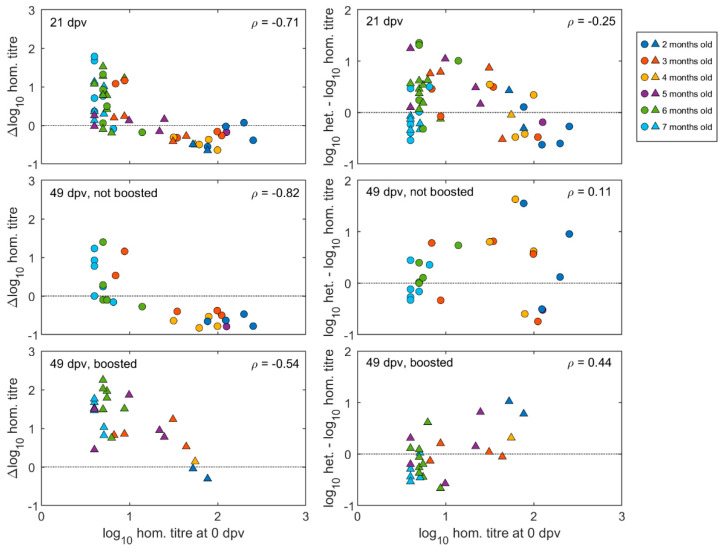
Effect of pre-vaccination homologous calf titres on post-vaccination antibody responses. Left hand panels show homologous titre changes from pre-vaccination values at given times, plotted against homologous pre-vaccination titres. Right hand panels show heterologous minus homologous log titres at given times, plotted against pre-vaccination homologous log titres. Age of animals (2–7 months) at vaccination according to colour coding in key, with circles representing unboosted calves and triangles representing calves given a booster vaccination at 21 dpv. In each panel, *ρ* gives Spearman’s rank correlation coefficient between the change in homologous titre or the difference in homologous and heterologous titres and the pre-vaccination titre.

Pre- and post-vaccination antibody titres of individual calves are illustrated in Figure 3 and Figure S1. The neutralising antibody responses at 21 days after the first vaccination were negatively correlated with the antibody titres at vaccination (Spearman’s rho = −0.71, *p* < 0.0001) (Figure 4). The final linear mixed model (Supplementary Table S1) indicated there was no significant (*p* > 0.05) increase in mean antibody titres after the first vaccination for animals with high levels of MDA in groups aged between two- and five-months-old, while there was a significant increase of around 1.0 log_10_ (95% CI: 0.6–1.6) for calves in the older age groups (at six and seven months of age). In 14 out of 20 calves vaccinated at less than five months old, the titres had reduced after three weeks, whereas for 24 out of 30 calves vaccinated from 5 months and older, the titres had increased (Figure 3 and Figure S1).

The effect of booster vaccination on calves with different levels of MDA were measured in sera collected at 49 days after the first vaccination, which was also 28 days after the booster for those that received it. Regardless of boosting, titres remained negatively correlated (Spearman’s rho = −0.74, *p* < 0.001) with those at vaccination (Figure 4).

Compared to levels at 21 dpv, 24 of 26 boosted calves showed an increase in VNT antibodies against the vaccine virus (A/May-97), irrespective of their age at first vaccination (Figure 3), with a mean increase in titre of 1.02 log_10_ (95% CI: 0.74–1.30) following the boost (Supplementary Table S2). Only one boosted calf had a VNT titre against the A/May-97 virus that was below the suggested minimum protective cut-off point (despite an increase after the booster vaccination) (Figure 3). By comparison, in 20 out of 24 unboosted calves, the titre fell between 21 and 49 dpv.

#### 3.2.2. Heterologous Neutralising Antibodies and Antigenic Match

Heterologous titres to A/MAY/2/2011 virus were lower than homologous titres in all the cows (Figure 1) and ranged from 0.82 to 2.70 (mean and standard deviation of 2.11 and 0.43). The estimated antigenic relationships (r_1_ values) between vaccine and field strain for sera from cows ranged from 0.01 to 0.59, with a mean of 0.25 (SD = 0.14), where 63% of animals had r_1_ values less than the suggested vaccine-match cut-off value of 0.3 [21,25]. Nevertheless, 90% of cows had log_10_ VNT titres against the heterologous A/MAY/2/2011 virus at or above the upper threshold of 1.67 log_10_ for 75% likelihood of cross-protection (Figure 1; ref. [24]) at the time of sampling.

The half-life for the decay of heterologous MDA was 3.40 (2.46–5.51) months, but levels were mostly below the 1.2 log_10_ threshold of expected protection. Most older calves had low pre-vaccination antibody titres to both strains. Point of vaccination sera from calves vaccinated at 2–4 months old showed a very similar pattern of antigenic discrimination to cows, with all calves having lower values to the heterologous virus resulting in r_1_ values ranging from 0.06 to 0.73 and a mean of 0.23 (SD = 0.19). Considering only 2–4-month-old calves with maternal titres ≥ 1.2 log_10_ to at least one virus tested for (i.e., unambiguously above background levels) the mean r_1_ value was 0.15 (SD = 0.07).

Patterns of neutralisation by sera collected from calves after vaccination showed variability in the degree to which the two viruses were most readily neutralised and for many sera, the heterologous virus was neutralised at a higher titre than the homologous virus (Figure 3), resulting in r_1_ values greater than 1 and up to 43 (Supplementary Table S2). For sera collected from calves after primary vaccination, the range of calculated r_1_ values was therefore much greater, at 0.10 to 23 (mean of 3.3, or 3.5 excluding sera with VNT values less than 1.2 log_10_), than for the cows and pre-vaccination calves. Most of the calves (57%) showed r_1_ values of more than 1.0, 35% calves showed r_1_ values above the suggested vaccine-match cut-off (0.3) and below 1.0, and only 9% showed r_1_ values below the suggested vaccine-match cut-off. Calves that received booster vaccination also showed high r_1_ values ranging from 0.18 to 43 (mean of 3.6, or 3.9 excluding sera with VNT values less than 1.2 log_10_); 42% had r_1_ values > 1.0 and 46% between 0.3 and 1.0.

When neutralisation titre differences between the two viruses at 49 dpv were plotted against the homologous pre-vaccination titres for the same calves, it could be seen for both boosted and un-boosted calves that relatively higher post-vaccination heterologous titres were correlated to homologous pre-vaccination antibodies. This effect was less apparent for sera collected after primary vaccination (i.e., 21 dpv) due to the presence of young calves that did not respond to the vaccination (Figure 4).

## 4. Discussion

Post-vaccination monitoring (PVM) studies provide important insights into the performance of FMD vaccines in the field. This study was conducted in Malaysia to assess the immune responses after vaccination with a commercially available FMD vaccine. Serotypes A immunity was studied as this is considered one of the most antigenically diverse.

Fifty-six days after their last vaccination, the mean VN titre of the cows to the homologous serotype A vaccine virus was 2.84 log_10_, which is above the 2.07 log_10_ titre threshold predicted for a 95% probability of homologous protection with serotype A [23]. The mean titre to the field strain A/MAY/2/2011 was 2.11 log_10_ which is above the 1.17–1.67 log_10_ titre range threshold predicted for a 75% probability of heterologous protection with different strains of serotype A [24]. Although the titres to the vaccine virus were always higher than those for the field virus, considerable variation in the relationship was evident, illustrating the difficulty in determining antigenic relationships with precision from studies employing a small number of antisera. As most of the cows appeared to likely be protected against the field virus, the study results also show how the use of a vaccine with a relatively poor antigenic match to the field virus (most cows had r_1_ values below the 0.3 threshold) can be compensated for by an appropriate combination of vaccine potency and vaccination regime [28]. There are conflicting reports on the extent to which repeated vaccination over several years can give rise to NSP seropositive results when a purified vaccine has been used [29,30]. In this study, where cows were bled 56 days after the last vaccination, none of the 48 animals showed NSP seroconversion after 7–22 vaccinations.

The levels of MDA were above the VN threshold for the expected 50% probability of homologous protection [23] in 16 out of 20 two-to-four-months-old calves prior to vaccination. Comparing the titres of pre-vaccination antibody in calves of different ages gave an estimate of 1.15 months (i.e., around 34.5 days) for antibody half-life. This can be compared with the 21–22 day period estimated from studies of antibody decay in unvaccinated calves and from which it was concluded that MDA will persist until calves reached 4–5 months of age (e.g., [16,31]), although this will also depend upon the immunity of dams and the amount of colostrum consumed by calves [32]. Due to the low antigenic match, the calves in this study were considered to have been unreliably protected against a heterologous challenge with A/MAY/2/2011, even at younger ages when less transferred antibodies had been lost. However, at the lowest possible threshold of 1.17 log_10_ determined for 75% serotype A cross-protection [24], some calves should have been protected up to around 3 months of age. If, as here, the same vaccine is used in the calves as the dams, this leaves a protection gap until responsive to revaccination [15]. In an effort to bridge this gap, the value of vaccinating calves with a heterologous strain compared to that given to their dams has been examined [31].

In this study of a well-vaccinated herd, these Malaysian calves became responsive to a first vaccination at ~5 months old. Therefore, the 2.5-month age cut-off age recommended for first vaccination in Malaysia seems too early for this type of farm if a single dose of vaccine is to be given. Responses were seen in calves of all ages after a booster given three weeks after the first vaccination, suggesting earlier vaccination could be effective when a second vaccine dose is given, but a longer follow-up of the responses would be needed to be sure of the correct ages for vaccination using this regime. A wider survey of additional vaccinated herds could reveal more about the levels and variability of immunity in cows and calves, which may be affected by the quality of the vaccines and the way that vaccination and colostrum feeding has been carried out. In a study of highly vaccinated dairy cattle in the Middle East, also using the Aftovaxpur vaccine and homologous VNT for serology, four vaccinations given monthly from 2.5 months of age was found to be optimal to avoid an immunity gap [33]. The variability of pre-vaccination calf titres despite feeding pooled colostrum was also noted, indicating that no single-dose vaccination regime will be optimal for all animals. Earlier work in similar farms [15] did not observe a priming effect when the vaccination was given early in the presence of MDA and the recommendation was to vaccinate three times at 4, 5, and 6 months of age. However, Nichols et al. (1984) [16] considered that calves with low MDA titres were able to be sensitized so that on revaccination they showed a satisfactory response, but MDA depressed responses to both primary and secondary vaccination. Based on their findings, a trial was undertaken of regularly vaccinated herds in Brazil, in which calf vaccination (given as two doses) was delayed until animals were 5–6 months old. This was said to have reduced FMD case numbers compared to farms where calves were vaccinated every four months, regardless of age. A system of first vaccinating calves at four months followed by boosting every three months until 18 months of age is sometimes used for prophylaxis with FMD vaccines produced in the Russian Federation [34].

Bucafasco et al. (2014) [10] reported that MDA prevented B cell responses but not T cell responses. In a subsequent study, they reported that young calves with MDA did not respond to a first dose of vaccine, but some responded to the booster [11]. Only animals with log_10_ VN titres < 2.0 responded at revaccination. But in all cases, antibodies were not sustained and weak compared to controls vaccinated without MDA. Çokçalışkan et al. (2017) [13] found that the VN titres of calves with MDA fell after vaccination but rebounded and expected protection was in some cases maintained especially after higher or double dose vaccination. They concluded that vaccinations should be delayed until calves are at least 3 months old and that they should be given a high potency vaccine, preferably with a booster after one month. Elnekave et al. (2016) [14] followed calves vaccinated three or four times (at 0, 4, 19 +/− 34 weeks) at ages up to 6 months for 70 weeks. Calves less than 3 months old responded relatively poorly, although a transient response was observed after a third and especially fourth vaccination; however, calves above 3 months old responded better. Some differences in the findings of these studies may be attributed to variation in the amount of MDA and in the adjuvants and potency of the vaccines used. In general, due to the many variables involved, as well as cost constraints, no single regime will be optimal in all situations.

Studies of MDA in relation to FMD have focused on homologous antibody responses, i.e., to the same strain(s) as in the vaccine. A novel aspect of the current study was the observation that MDA was associated with enhancement of the response to a heterologous virus. The antigenic relationship (r_1_) between the serotype A strain of virus included in the vaccine (A May 2007) and the field strain under study (A/MAY/2/2011) averaged at 0.23 and 0.25 using sera from pre-vaccinated calves and cows, respectively. Sera from some animals gave higher or lower relationships, likely reflecting differences in epitope recognition preferences, but the sera from all 68 cattle for which r_1_ was estimated neutralised the homologous virus more efficiently than the heterologous one. This was in marked contrast to sera from calves after vaccination in the presence of MDA, when many calves developed elevated responses to the heterologous virus, even when titres sometimes fell against the homologous virus. Bucafasco et al. (2014) [10] suggested that masking of VN epitopes by MDA may reduce active VN responses and epitope masking [35,36,37] is a possible explanation for the enhanced heterologous responses. An explanatory hypothesis for the results here could be that passive antibodies mask strain-specific dominant epitopes when vaccine is given in the presence of MDA resulting in antibody responses to sub-dominant epitopes that are more cross-reactive (conserved). This could account for improved cross-neutralization of a heterologous strain by sera from vaccinated calves, but not for post-vaccination sera of calves with MDA having greater heterologous neutralization than homologous neutralization. An explanation for this effect could be that antibodies to conserved epitopes are better “virus neutralizers” but get blocked from binding by homologous strain-specific antibodies to dominant (“decoy”) epitopes that do not bind to heterologous virus. A question remains as to why, if passive antibodies are masking epitopes in calves, it does not do so when it is actively acquired in revaccinated adults.

There were insufficient sera to do additional testing in order to measure the breadth of antigenic coverage against further viruses of serotype A and other serotypes. Further studies are warranted to examine the nature of the cross-reactivity of antibody responses from more cattle when vaccinated in the presence of passively acquired antibodies in order to confirm these unexpected findings and to better understand the implications for the protection of vaccinated calves and for broadening the antibody response to FMD vaccinations in other situations.

## 5. Conclusions

The main findings of the study were that cows regularly vaccinated with a purified FMD vaccine developed neutralising antibodies at high titre against the homologous vaccine strains. Despite a borderline antigenic match to a heterologous field virus, the titres were most likely high enough to be protective against this strain when measured 56 days after vaccination. The vaccine was of sufficient purity that none of the 48 cows developed NSP antibodies, even after up to 22 vaccinations. Calves born to such well-vaccinated cows did not respond reliably to vaccination at 2–5 months of age, and even 6–7 month-old calves did not always sustain post-vaccination responses between 21 and 49 days after vaccination. A second dose of vaccine given 3 weeks after the primary dose improved antibody responses in all age groups three weeks after the second dose had been given, but to what extent the induced antibodies persisted thereafter was not determined. Due to situation-specific variables, such as vaccine quality and vaccination regime, there is a need to monitor the levels of immunity in cows and their calves to help select the best time for calfhood vaccination. The most novel finding of this study was that calf vaccination variably enhanced heterologous antibody responses, the effect being corelated to the strength of the homologous titre at vaccination. This could be due to epitope masking by passively acquired maternal antibody and requires further investigation. Firmer recommendations on vaccination regimes for calves require longer follow-up to clarify the duration of the post-vaccination antibody responses and to confirm the impact of maternal antibodies on the antigenic specificity of the immune response.

## Data Availability

Data provided in Supplementary Tables.

## Supplementary Materials

The following supporting information can be downloaded at: https://www.mdpi.com/article/10.3390/vaccines11121844/s1, Table S1: Final linear mixed model for homologous and heterologous log_10_ virus neutralising titres against foot-and-mouth disease virus in calves following vaccination. Table S2: Virus neutralising antibody titres and associated r_1_ values for cows and calves. Figure S1: Pre- and post-vaccination homologous and heterologous log_10_ VN titres of calves in different age groups.

## References

[1] Thomson G.R., Bastos A.D.S., Coetzer J.A.W., Tustin R.C. (2004). Foot-and-mouth disease. Infectious Diseases of Livestock.

[2] Knight-Jones T.J., Rushton J. (2013). The economic impacts of foot and mouth disease—What are they, how big are they and where do they occur?. Prev. Vet. Med..

[3] Leforban Y., Gerbier G. (2002). Review of the status of foot and mouth disease and approach to control/eradication in Europe and Central Asia. Rev. Sci. Tech..

[4] Saraiva V., Darsie G. (2004). The use of vaccines in South American foot-and-mouth disease eradication programmes. Dev. Biol..

[5] Paton D.J., Sumption K.J., Charleston B. (2009). Options for control of foot-and-mouth disease: Knowledge, capability and policy. Philos. Trans. R. Soc. Lond. B Biol. Sci..

[6] Ferrari G., Paton D., Duffy S., Bartels C., Knight-Jones T., Metwally S., Münstermann S. (2016). Foot and Mouth Disease Vaccination and Post-Vaccination Monitoring: Guidelines.

[7] Pay T.W., Kurstak E. (1984). Factors influencing the performance of foot-and-mouth disease vaccines under field conditions. Applied Virology.

[8] Knight-Jones T.J., Bulut A.N., Gubbins S., Stark K.D., Pfeiffer D.U., Sumption K.J., Paton D.J. (2015). Randomised field trial to evaluate serological response after foot-and-mouth disease vaccination in Turkey. Vaccine.

[9] Parida S. (2009). Vaccination against foot-and-mouth disease virus: Strategies and effectiveness. Expert Rev. Vaccines.

[10] Bucafusco D., Di Giacomo S., Pega J., Juncos M.S., Schammas J.M., Pérez-Filgueira M., Capozzo A.V. (2014). Influence of antibodies transferred by colostrum in the immune responses of calves to current foot-and-mouth disease vaccines. Vaccine.

[11] Bucafusco D., Pereyra R., Mansilla F.C., Malacari D.A., Juncos M.S., Di Giacomo S., Ayude A.F., Pérez-Filgueira M., Capozzo A.V. (2019). Immune cells transferred by colostrum do not influence the immune responses to foot-and-mouth disease primary vaccination. J. Dairy. Sci..

[12] Chase C.C., Hurley D.J., Reber A.J. (2008). Neonatal immune development in the calf and its impact on vaccine response. Vet. Clin. N. Am. Food Anim. Pract..

[13] Çokçalışkan C., Türkoğlu T., Uzunlu E., Sareyyüpoğlu B., Hancı I., İpek A., Arslan A., Babak A., İldeniz G., Gülyaz V. (2017). Influence of vaccine potency and booster administration of foot-and-mouth disease vaccines on the antibody response in calves with maternal antibodies. J. Vet. Sci..

[14] Elnekave E., Dekker A., Eble P., van Hemert-Kluitenberg F., Gelman B., Storm N., Klement E. (2016). The long term effect of age and maternally derived antibodies against foot and mouth disease on the serological response following vaccination in young dairy calves. Vaccine.

[15] Kitching R.P., Salt J.S. (1995). The interference by maternally-derived antibody with active immunization of farm animals against foot-and-mouth disease. Br. Vet. J..

[16] Nicholls M.J., Black L., Rweyemamu M.M., Genovese J., Ferrari R., Hammant C.A., de Silva E., Umehara O. (1984). The effect of maternally derived antibodies on the response of calves to vaccination against foot and mouth disease. J. Hyg..

[17] Patil P.K., Sajjanar C.M., Natarajan C., Bayry J. (2014). Neutralizing antibody responses to foot-and-mouth disease quadrivalent (type O, A, C and Asia 1) vaccines in growing calves with pre-existing maternal antibodies. Vet. Microbiol..

[18] Sadir A.M., Schudel A.A., Laporte O., Braun M., Margni R.A. (1988). Response to foot-and-mouth disease vaccines in newborn calves. Influence of age, colostral antibodies and adjuvants. Epidemiol. Infect..

[19] Abdul-Hamid N.F., Hussein N.M., Wadsworth J., Radford A.D., Knowles N.J., King D.P. (2011). Phylogeography of foot-and-mouth disease virus types O and A in Malaysia and surrounding countries. Infect. Genet. Evol..

[20] Sørensen K.J., Madsen K.G., Madsen E.S., Salt J.S., Nqindi J., Mackay D.K. (1998). Differentiation of infection from vaccination in foot-and-mouth disease by the detection of antibodies to the non-structural proteins 3D, 3AB and 3ABC in ELISA using antigens expressed in baculovirus. Arch. Virol..

[21] (2023). Foot-and-mouth disease. Manual of Diagnostic Tests and Vaccines for Terrestrial Animals.

[22] Vianna Filho Y.L., Astudillo V., Gomes I., Fernandez G., Rozas C.E., Ravison J.A. (1993). Potency control of foot-and-mouth disease vaccine in cattle. Comparison of the 50% protective dose and the protection against generalization. Vaccine.

[23] Barnett P.V., Statham R.J., Vosloo W., Haydon D.T. (2003). Foot-and-mouth disease vaccine potency testing: Determination and statistical validation of a model using a serological approach. Vaccine.

[24] Gubbins S., Paton D.J., Dekker A., Ludi A.B., Wilsden G., Browning C.F.J., Eschbaumer M., Barnabei J., Duque H., Pauszek L.L. (2022). Predicting cross-protection against foot-and-mouth disease virus strains by serology after vaccination. Front. Vet. Sci..

[25] Rweyemamu M.M. (1984). Antigenic variation in foot-and-mouth disease: Studies based on the virus neutralization reaction. J. Biol. Stand..

[26] Pinheiro J., Bates D., R Core Team (2023). Nlme: Linear and Nonlinear Mixed Effects Models. R Package Version 3.1-162. https://CRAN.R-project.org/package=nlme.

[27] R Core Team (2023). R: A Language and Environment for Statistical Computing.

[28] Brehm K.E., Kumar N., Thulke H.H., Haas B. (2008). High potency vaccines induce protection against heterologous challenge with foot-and-mouth disease virus. Vaccine.

[29] Mackay D.K.J., Forsyth M.A., Davies P.R., Berlinzani A., Belsham G.J., Flint M., Ryan M.D. (1998). Differentiating infection from vaccination in foot-and-mouth disease using a panel of recombinant, non-structural proteins in ELISA. Vaccine.

[30] Smitsaart E., Espinoza A.M., Maradei E., Cosentino B., Guinzburg M., Madonni G., Cadenazzi G., Bottini R., Filippi J., Bergmann I. (2015). Importance of foot and mouth disease vaccine purity in interpreting serological surveys. Rev. Sci. Tech..

[31] Dekker A., Eble P., Stockhofe N., Chenard G. (2014). Intratypic heterologous vaccination of calves can induce an antibody response in presence of maternal antibodies against foot-and-mouth disease virus. BMC Vet. Res..

[32] Sareyyüpoğlu B., Gülyaz V., Çokçalışkan C., Ünal Y., Çökülgen T., Uzunlu E., Gürcan S., İlk O. (2019). Effect of FMD vaccination schedule of dams on the level and duration of maternally derived antibodies. Vet. Immunol. Immunopathol..

[33] Hamers C., Broks V., Giskus P., Alnahwary M., Denormandie N., Hudelet P. Maternally Derived Antibodies to FMD in Cattle: Is Interference on FMD Vaccination Appropriately Considered?. Proceedings of the Poster. 2020 Open Session of the Standing Technical Committee of the EuFMD.

[34] Kharatyan S., Sargsyan K., Elbakyan H., Markosyan T., Tumanyan P., Hakobyan V., Sargsyan V., Badalyan M., Chobanyan G., Achenbach J.E. (2023). Evaluation of the effectiveness of foot-and-mouth disease vaccination of animals in the buffer zone of the Republic of Armenia in 2016–2020. BMC Vet. Res..

[35] Bergström J.J., Xu H., Heyman B. (2017). Epitope-Specific Suppression of IgG Responses by Passively Administered Specific IgG: Evidence of Epitope Masking. Front. Immunol..

[36] McNamara H.A., Idris A.H., Sutton H.J., Vistein R., Flynn B.J., Cai Y., Wiehe K., Lyke K.E., Chatterjee D., Kc N. (2020). Antibody Feedback Limits the Expansion of B Cell Responses to Malaria Vaccination but Drives Diversification of the Humoral Response. Cell Host Microbe.

[37] Meyer-Hermann M. (2019). Injection of Antibodies against Immunodominant Epitopes Tunes Germinal Centers to Generate Broadly Neutralizing Antibodies. Cell Rep..

