# Supplementary material for: Maternally Derived Antibodies to Foot-and-Mouth Disease Virus Modulate the Antigenic Specificity of Humoral Responses in Vaccinated Cattle
Source: Vaccines (Basel). 2023 Dec 13;11(12):1844. doi: 10.3390/vaccines11121844 (PMC10747493; doi:10.3390/vaccines11121844)
Supplement: Supplementary file 1 [file vaccines-11-01844-s001.zip › FigS1_calfVNT.pptx]

## Slide 1
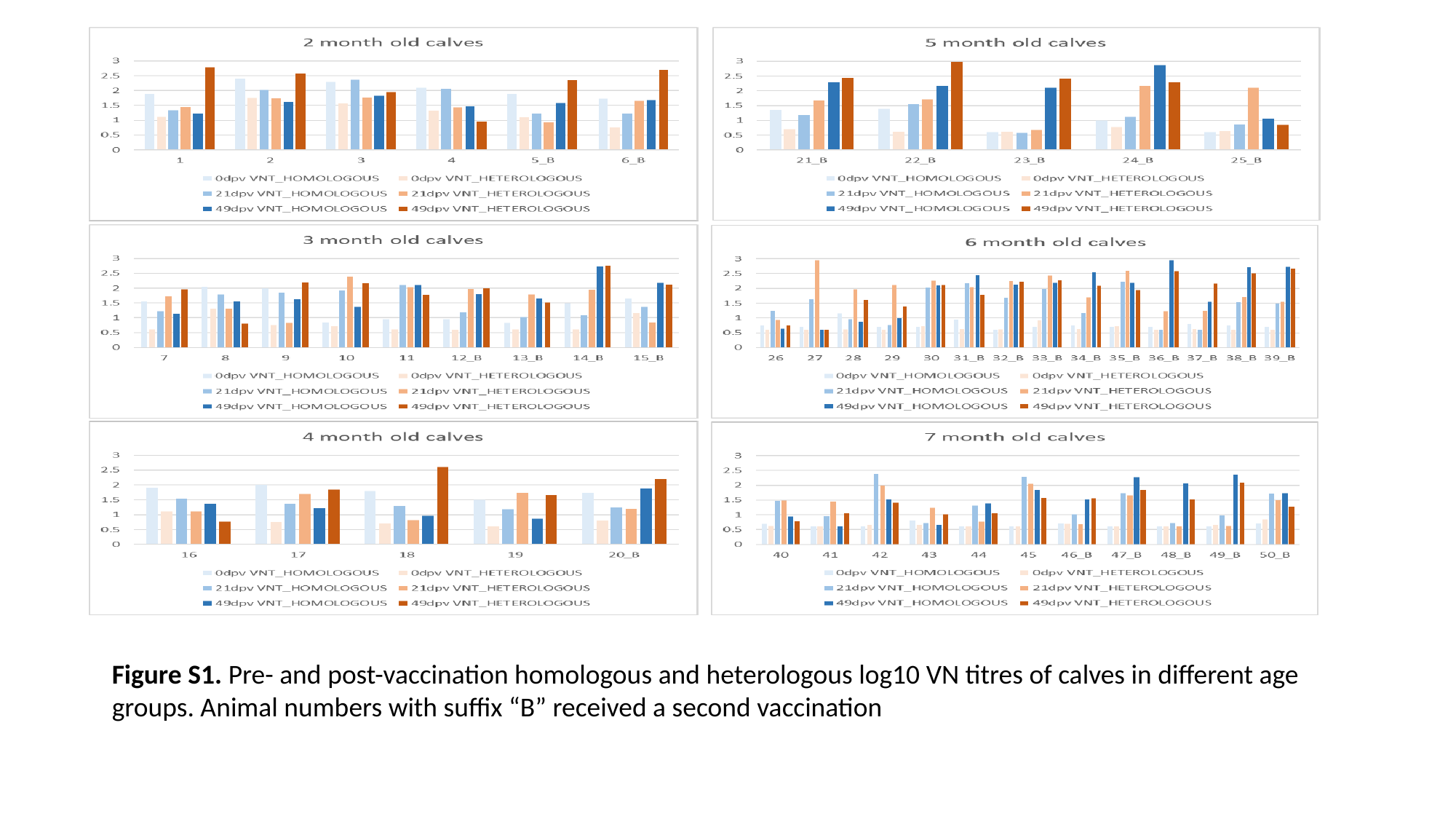

Figure S1. Pre- and post-vaccination homologous and heterologous log10 VN titres of calves in different age groups. Animal numbers with suffix “B” received a second vaccination
