# Supplementary material for: Maternally Derived Antibodies to Foot-and-Mouth Disease Virus Modulate the Antigenic Specificity of Humoral Responses in Vaccinated Cattle
Source: Vaccines (Basel). 2023 Dec 13;11(12):1844. doi: 10.3390/vaccines11121844 (PMC10747493; doi:10.3390/vaccines11121844)
Supplement: Supplementary file 1 [file vaccines-11-01844-s001.zip › SupplementaryTableS1_model.pdf]

Supplementary Table S1. Final linear mixed model for homologous and heterologous log_{10} virus neutralising titres against foot-and-mouth disease virus in calves following vaccination.

coefficient	homologous titres			heterologous titres		
	estimate	s.e.*	*p*-value	estimate	s.e.*	*p*-value
intercept	2.26	0.19	<0.001	1.36	0.20	<0.001
age (months)						
2	baseline	-	-	baseline	-	-
3	-0.71	0.24	0.006	-0.55	0.26	0.04
4	-0.45	0.26	0.09	-0.55	0.28	0.04
5	-0.50	0.35	0.17	-0.90	0.36	0.02
6	-1.61	0.24	<0.001	-0.77	0.25	0.003
7	-1.55	0.23	<0.001	-0.65	0.25	0.01
dpv						
0	baseline	-	-	baseline	-	-
21	-0.31	0.22	0.16	0.29	0.26	0.27
49	-0.82	0.22	<0.001	0.72	0.26	0.007
boost						
no	baseline	-	-	baseline	-	-
yes	-0.64	0.25	0.02	-0.30	0.25	0.24
age:dpv†						
3 - 21	0.49	0.27	0.07	0.66	0.33	0.05
4 - 21	-0.14	0.31	0.66	0.26	0.38	0.49
5 - 21	0.42	0.31	0.17	0.86	0.37	0.02
6 - 21	1.04	0.26	<0.001	1.10	0.31	<0.001
7 - 21	1.09	0.26	<0.001	0.42	0.32	0.19
3 - 49	0.79	0.27	0.004	0.11	0.33	0.74
4 - 49	0.09	0.31	0.76	0.15	0.38	0.68
5 - 49	0.76	0.31	0.02	-0.03	0.37	0.94
6 - 49	1.31	0.26	<0.001	0.08	0.31	0.79
7 - 49	1.24	0.26	<0.001	-0.32	0.32	0.32
age:boost†						
3 - yes	0.22	0.30	0.47	0.20	0.28	0.47
4 - yes	0.54	0.39	0.17	0.24	0.36	0.52
5 - yes	-0.07	0.38	0.86	0.55	0.36	0.13
6 - yes	0.81	0.28	0.006	0.39	0.26	0.15
7 - yes	0.51	0.29	0.09	0.15	0.27	0.59
boost:dpv†						
21 - yes	-0.10	0.15	0.51	-0.17	0.19	0.35
49 - yes	1.02	0.15	<0.008	0.71	0.19	<0.001

* s.e. - standard error

† interaction term
